# Immunotherapy Goes Local: The Central Role of Lymph Nodes in Driving Tumor Infiltration and Efficacy

**DOI:** 10.3389/fimmu.2021.643291

**Published:** 2021-03-01

**Authors:** Kim M. van Pul, Marieke F. Fransen, Rieneke van de Ven, Tanja D. de Gruijl

**Affiliations:** ^1^Department of Medical Oncology, Amsterdam University Medical Centers, Cancer Center Amsterdam, Amsterdam Infection and Immunity Institute, Vrije Universiteit Amsterdam, Amsterdam, Netherlands; ^2^Deparment of Pulmonary Diseases Amsterdam University Medical Centers, Cancer Center Amsterdam, Amsterdam Infection and Immunity Institute, Vrije Universiteit Amsterdam, Amsterdam, Netherlands; ^3^Department of Otolaryngology/Head-Neck Surgery, Amsterdam University Medical Centers, Cancer Center Amsterdam, Amsterdam Infection and Immunity Institute, Vrije Universiteit Amsterdam, Amsterdam, Netherlands

**Keywords:** cancer, tumor draining lymph node, dendritic cell, immune check point, immune exclusion, t cell exhaustion, CTLA-4, PD-1

## Abstract

Immune checkpoint blockade (ICB) has changed the therapeutic landscape of oncology but its impact is limited by primary or secondary resistance. ICB resistance has been related to a lack of T cells infiltrating into the tumor. Strategies to overcome this hurdle have so far focused on the tumor microenvironment, but have mostly overlooked the role of tumor-draining lymph nodes (TDLN). Whereas for CTLA-4 blockade TDLN have long since been implicated due to its perceived mechanism-of-action involving T cell priming, only recently has evidence been emerging showing TDLN to be vital for the efficacy of PD-1 blockade as well. TDLN are targeted by developing tumors to create an immune suppressed pre-metastatic niche which can lead to priming of dysfunctional antitumor T cells. In this review, we will discuss the evidence that therapeutic targeting of TDLN may ensure sufficient antitumor T cell activation and subsequent tumor infiltration to facilitate effective ICB. Indeed, waves of tumor-specific, proliferating stem cell-like, or progenitor exhausted T cells, either newly primed or reinvigorated in TDLN, are vital for PD-1 blockade efficacy. Both tumor-derived migratory dendritic cell (DC) subsets and DC subsets residing in TDLN, and an interplay between them, have been implicated in the induction of these T cells, their imprinting for homing and subsequent tumor control. We propose that therapeutic approaches, involving local delivery of immune modulatory agents for optimal access to TDLN, aimed at overcoming hampered DC activation, will enable ICB by promoting T cell recruitment to the tumor, both in early and in advanced stages of cancer.

## Introduction

Over the past decade, it has become clear that for immune checkpoint blockade (ICB) to work, tumors need to contain sufficient numbers of infiltrating T cells (1, 2). Particularly in view of the perceived mechanism-of-action of PD-1 inhibitors this would make sense, since it is supposed to entail the release of cancer-imposed brakes from tumor-infiltrating cytotoxic effector T cells. An intense research effort has therefore been ongoing to characterize the tumor microenvironment (TME) and find ways to ensure T-cell infiltration (3, 4). New insights point to the need for therapeutic targeting of tumor-draining lymph nodes (TDLN), rather than of the TME, to secure proper antitumor T-cell generation and at the same time brisk tumor infiltration (5–8). TDLN can either be more proximal or more distal from the tumor, but they are all part of the lymph catchment area of the tumor. As a result of this definition, non-TDLN may sometimes be more proximal to the tumor than TDLN, but due to the fact that tumor-derived factors will diffuse through the lymph basin, be less affected by the tumor, e.g., in terms of immune suppression (9). A growing number of studies are exploring the use of systemically administered immune checkpoint inhibitors (ICI) as neo-adjuvant therapy for patients in earlier (i.e., resectable) cancer stages (10–13). As in this setting both the primary tumor and TDLN are still in place (rather than surgically removed in the adjuvant setting) this approach will enable simultaneous immune modulation of the TME and of TDLN. As a result, these studies are generating a renewed interest in the contribution of TDLN to the efficacy of ICB. We and others have shown in pre-clinical models that TDLN play a pivotal role in PD-1/PD-L1 blocking antibody therapy, and that surgical resection of TDLN prior to treatment hampers therapeutic outcome (5, 14). In pre-clinical models, lymphatic drainage has also been shown to facilitate the priming of anti-tumor T-cell immunity (15, 16). Indeed, recent evidence points to the need for the recruitment to the tumor of newly primed and peripherally (e.g., in TDLN) expanded effector T cells to ensure efficacy of ICB (17). Clinical efficacy and durability of antitumor immunity appears to be associated with elevated frequencies of central-memory or early-effector T cells with the ability to home to lymph nodes (18–20). More in-depth knowledge on the exact nature of the T cells amenable to ICB and the underlying molecular mechanisms that control their activation, point to the importance of Dendritic Cells (DC) in driving waves of newly primed or reinvigorated early-effector T cells to facilitate effective ICB (7, 21–23). In this review we will discuss mechanisms underlying tumor-associated immune suppression of TDLN and how we can use this knowledge to devise new local intervention strategies aimed at harnessing TDLN to secure efficacy of cancer immunotherapy, both in early and in advanced stages of cancer development.

## Immune Suppression of Dendritic Cells in TDLN: Early Immune Escape

**Figure 1:**
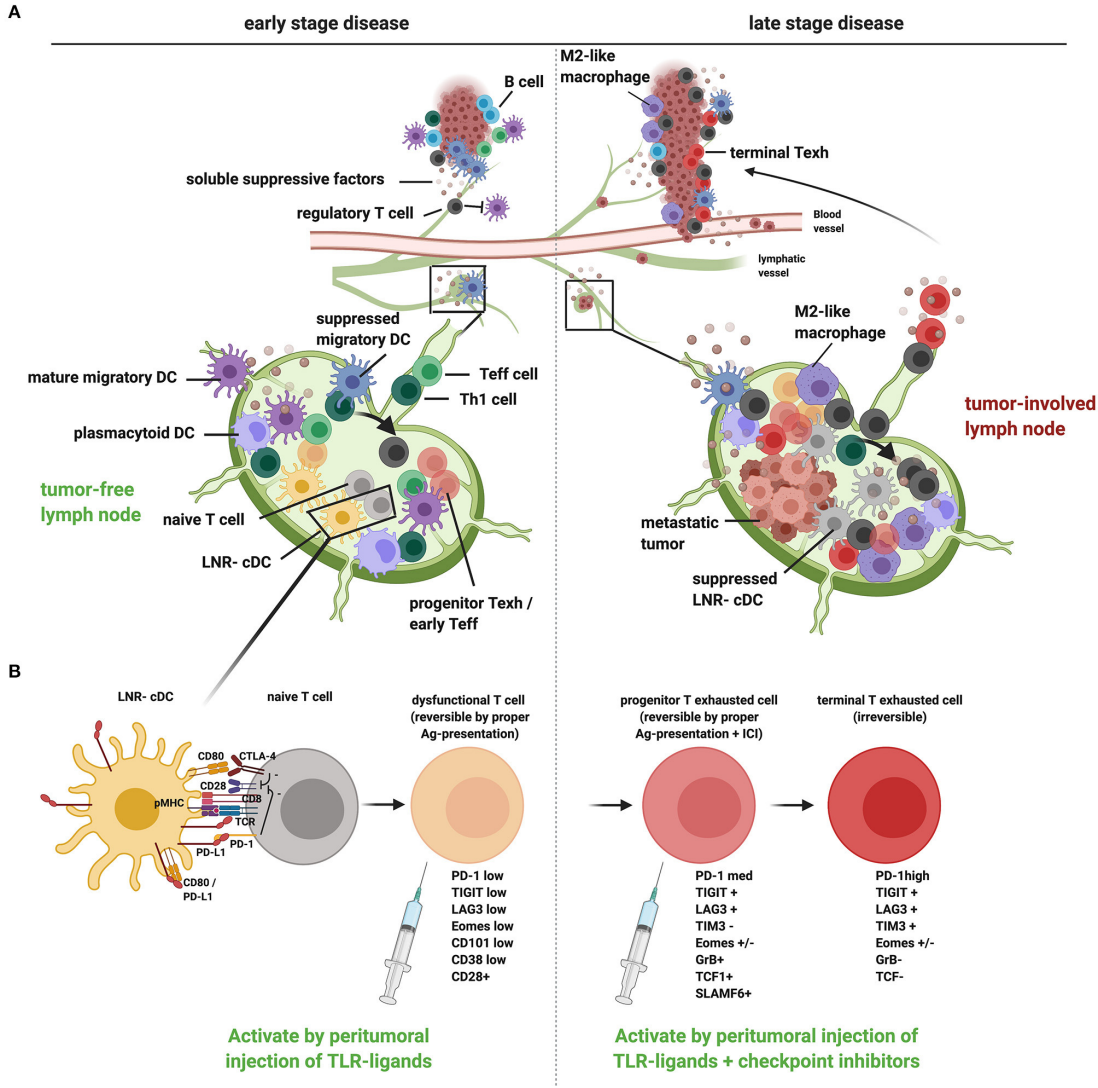
A proposed model of tumor-induced immune suppression of draining lymph nodes and local therapeutic intervention opportunities to overcome T cell dysfunction and exclusion in early- and late-stage cancer development. **(A)** Schematic representation of how tumors, at early (left) and late stage (right), suppress the loco-regional immune response in the tumor as well as in the tumor-draining lymph nodes (TDLN). In early-stage disease, migratory DCs are hampered in their migration and activation [through release of suppressive factors in the tumor microenvironment (TME) and interactions with Tregs], resulting in suboptimal T cell priming and activation in the TDLN (generating dysfunctional T cells), conversion of Th cells to Tregs (see arrow), and reduced recruitment of Teff cells to the tumor. In late-stage disease, upon metastatic spread to the TDLN, LNR-cDC are profoundly suppressed, leading to the priming and expansion of dysfunctional progenitor exhausted T cells and Tregs in TDLN and poor recruitment of Teff to the tumor. Active suppression in the TME (with accumulating myeloid regulatory cells like M2 macrophages and a lack of mature DCs) contributes to the differentiation of terminally exhausted T cells and Treg recruitment with possible immune exclusion. **(B)** Suboptimal priming in the TDLN due to suppression of cDC, accompanied by excess PD-L1 surface expression, results in restrained T cell priming and deviated CD8+ T cell differentiation, marked by a reversibly dysfunctional state in early cancer development. Chronic high-dose (neo)-antigen stimulation in later stages of cancer development and progression will lead to the development of progenitor-exhausted and, ultimately, terminally exhausted T cells, marked by progressively higher PD-1 expression levels and the co-expression of other immune checkpoints, like LAG3, TIM3, and TIGIT. Typical markers for the different stages of dysfunction/exhaustion are listed (25–27). Whereas progenitor exhausted T cells can be rescued by immune checkpoint blockade, terminal exhaustion is an irreversible state due to epigenetic programming. Local immunotherapy, targeted at TDLN conditioning, can restore the anti-tumor T cell response by promoting DC activation (e.g., through local injection of TLR-L): in early cancer stages without tumor involvement of TDLN this may suffice to reverse T cell dysfunction and kick-start effective systemic antitumor immunity. In advanced cancer additional immune checkpoint blockade in the TDLN will enable reinvigoration of progenitor exhausted T cells, which can then home to the tumor and populate the TME, thus overcoming immune exclusion. This image was created using Biorender.com.

TDLN represent the site where T cells will first be primed against tumor-associated (neo)antigens. In order to escape the immune response, it is vital for tumors to nip this induction of tumor reactive T cells in the bud. The more immunogenic the tumor, the more pressing this matter becomes. With a high mutational burden, melanoma is the most immunogenic tumor type identified to date (24). As tumors develop, the cellular content of their TDLN shifts (Figure 1A). In breast, melanoma and cervical TDLN shifts in CD4/CD8 T cell ratios and elevated Treg rates were observed prior to metastatic involvement, but even more pronounced after (28–30). As metastases in the TDLN grow, memory T cell rates grow and myeloid regulatory cells are recruited (29, 30). Already at early stages of melanoma development, the primary tumor exerts an immunosuppressive effect on its TDLN through the release of immune modulatory exosomes and soluble mediators, which can ultimately lead to a “tumor-supportive” microenvironment, i.e., the pre-metastatic niche (31). In the first-line draining TDLN, the so-called sentinel lymph node (SLN), we have found clear evidence of early suppression of DC (28, 29). DC subsets in TDLN encompass migratory conventional DC (cDC) subsets (marked by CD1a expression in human epithelium draining lymph nodes) as well as lymph node-resident cDC (LNR-cDC, marked by high CD11c levels, various CD1c, CD141, and CD14 expression patterns, and absence of CD1a) and plasmacytoid DC (pDC; CD11c^−^CD123^hi^CD303^+^). Recent studies have shown the *in-vivo* exchange of antigens between migratory cDC and LNR-cDC and have demonstrated their concerted and coordinated activities to lead to optimal priming of an effective antitumor T-cell response (32–34). Whereas significantly lower levels of maturation and co-stimulatory markers were found in migratory cDC subsets already in Stage-2 melanoma, expression of these markers dropped profoundly in LNR-cDC only by Stage-3 (28). A significant negative correlation between the frequency and activation state of migratory cDC subsets in melanoma SLN and primary tumor size (Breslow thickness), suggested that the developing primary melanoma created a pre-metastatic niche in the TDLN by suppressing the migration of antigen-carrying cDC from the tumor to the TDLN. This early reduction in frequency of migratory cDC is consistent with observations made in murine models by Binnewies et al. (35) who reported that cDC2 migration from the tumor to TDLN was constrained by regulatory T cells (Tregs) in the tumor, resulting in suboptimal priming of Th cells and their failure to migrate to the tumor in sufficient numbers to support an anti-tumor response. In human melanoma SLN, metastasis size was inversely correlated with the frequency and activation state of LNR-cDC in SLN (28). Remarkably, whereas reduced frequencies of migratory cDC subsets was related to decreased local recurrence-free survival (RFS), reduced activation of LNR-cDC was related to decreased distant RFS (28). This suggests differential imprinting for homing properties of tumor-specific T cells by specific cDC subsets, and indicates an essential role for LNR-cDC in the induction of effective systemic antitumor immunity. In breast cancer SLN a similar progressive reduction in the activation state of LNR-cDC was observed, which was most pronounced upon metastatic involvement and then coincided with increased Treg rates, high co-expression levels of CTLA-4 and PD-1, and profoundly suppressed effector-T cell activity in the SLN (29). A possible role for LNR-cDC in keeping PD-1+ T cells in check was suggested by our finding that LNR-cDC in early-stage melanoma SLN expressed relatively higher PD-L1 levels as compared to CD80 (36). This indicates an inability of CD80 to keep PD-L1 from interacting with PD-1 on T cells through in-cis interactions (23, 37) and would fit with LNR-cDC subsets restraining antitumor T cells in early stages of cancer in a PD-1 dependent manner as recently reported (22). Dammeijer et al. (7) showed an association between poor RFS with high frequencies of PD-1/PD-L1 interactions between T cells and cDCs in SLN from Stage-2 melanoma patients. In addition, we found a strong inverse correlation between the activation state of LNR-cDC and Treg rates in melanoma SLN (28). This increase of Tregs accompanying decreased LNR-cDC activation may be responsible for subsequent T-cell anergy induction and the conversion of Ag-specific naïve T cells into Tregs in TDLN, as described by Alonso et al. (38) in a lung adenocarcinoma mouse model. Polychromatic FACS analysis showed CLEC9A+ LNR-cDC to consist mostly of cDC2 expressing both CD1c and intermediate levels of CD141 and of a minority of CD141hi cDC1 (39, 40). Their superior cross-presentation and -priming ability and their apparent relationship to the generalized immune state of the SLN and distant RFS, make LNR-cDC attractive targets for early therapeutic intervention to curb metastatic spread and outgrowth (36).

## Therapeutic Targeting of Early-Stage TDLN: “Think Global, Act Local”

We have obtained clinical evidence that local administration (i.e., intradermal injection around the primary tumor excision site) of immune modulatory agents in early-stage melanoma, aimed at TDLN immune potentiation, leads to systemic antitumor immune activation and increased RFS. In two randomized Phase-II trials in clinical stage-1/2 melanoma patients, we have shown that this intradermal administration of one or two doses of the TLR9 agonist CPG7909 (CpG-B), with or without GM-CSF, in the week leading up to the SLN procedure, resulted in enhanced LNR-cDC activation and melanoma antigen-specific T-cell responses in both the SLN and in peripheral blood (39, 41–43). Clinical analysis of the 52 patients participating in these trials showed a significantly and profoundly lower number of tumor positive SLN and (at a median follow-up of 88.8 months) a significantly increased RFS in patients receiving CpG-B as compared to patients receiving a saline placebo (44). *In vitro*, cultures of single-cell suspensions of breast cancer SLN with CpG-B similarly showed enhanced LNR-cDC activation and increased expression of effector T-cell-recruiting chemokines and cytokines associated with a Th1 response (40). The addition of a JAK2/STAT3 inhibitor interfered with negative feedback loops activated by CpG-B, resulting in further enhanced DC activation, down-regulated Th2 rates, and constrained Treg expansion (40). Altogether, these observations are consistent with the reinvigoration and boosting of pre-existent but dysfunctional T cells in TDLN, through the activation of LNR-cDC, providing protection against metastatic spread and outgrowth (see Figure 1). This is consistent with findings from a previous study by Schietinger et al. (25) showing that *in-vivo* antigen-driven T-cell dysfunction in early developing tumors is reversible. These clinical studies have clearly demonstrated the systemic immune activating effects of locally administered immune modulatory agents, resulting in long-term protection against loco-regional as well as distant metastases. Moreover, they have delivered important proof-of-concept that in the absence of the primary tumor (but presence of TDLN), direct immune modulation of the TDLN can lead to effective systemic antitumor immunity.

## Overcoming Immune Exclusion by Targeting DC in TDLN: Reinvigoration of Exhausted T Cells

While reinvigoration of suppressed T cells in early tumor stages may only require the “pushing of the gas pedal” by delivering DC-activating agents to TDLN, in more advanced stages simultaneous “lifting of the brakes” may be required by immune checkpoint blockade (Figure 1B). Recent insights hold that effective ICB would require the reinvigoration of so-called exhausted CD8+ T cells (7, 26, 45), which are regarded as a T-cell lineage that usually arises through chronic stimulation with high antigen doses (18). Exhausted T cells display loss of effector functions, accompanied by high expression levels of PD-1 in concert with multiple immune checkpoints. This exhausted phenotype has been linked to the activation of specific epigenetic regulatory programs (46). In cancer, exhausted T cells have been identified, which are replenished from a proliferative pool of so-called stem cell-like or progenitor-exhausted T cells: these progenitors are typified by intermediate surface levels of PD-1 and their expression of TCF-1 and SLAMF6. Recent findings show that in contrast to terminally exhausted T cells, these progenitor-exhausted T cells are still amenable to PD-1 blockade and as such may represent the prime targets for PD-(L)1 checkpoint inhibition (26). Other studies have pointed to CD8+ stem cell–like T cells or early-effector T cells as the foremost targets for effective PD-1 blockade (26, 45, 47–49). These populations, which may in part overlap, have been characterized as having a preserved capacity for proliferation and the ability to exert polyfunctional effector functions (26). Importantly, they are also commonly distinguished by their expression of CD28. Indeed, CD28 was shown to be required for effective PD-1 inhibition (48, 50). This is remarkable and points to the need for CD80/CD86 co-stimulation in addition to the “mere” interruption of PD-1 binding to its ligands PD-L1 and PD-L2 in order to unleash the full force of an antitumor effector T-cell response. This is in keeping with the observation of proliferative tumor-infiltrating CD8+ T cells upon clinical PD-1 blockade (1, 45). These proliferating T cells have a stem-cell phenotype and are found in niches with cDCs (51), which can provide CD80/CD86 co-stimulation. A recent study by Oh and colleagues showed that rather than tumor-expressed PD-L1, PD-L1 expression by infiltrating and cross-presenting DCs dictated PD-1 blockade efficacy (23). Similarly, Garris et al. (21) demonstrated that full-fledged activation of antitumor T cells by anti-PD-1 involved T-cell-DC crosstalk and was licensed by IFNγ and IL-12. This is all the more remarkable since macrophages by far outnumber DCs in tumors, and may be due to the fact that DCs, in contrast to tumor-associated macrophages (TAMs), express CD80. CD80 interacts with PD-L1 in-cis (37), resulting in a block of PD-1 binding to PD-L1 with maintained CD80 co-stimulatory activity through interactions with CD28 on progenitor-exhausted, early-effector or stem-cell like T cells. Indeed, the importance of DCs for PD-1 inhibition efficacy *in vivo* was recently linked to the relative expression levels of PD-L1 and CD80, which were shown to dictate T-cell priming efficacy of DCs (22). This finding was confirmed by relatively high levels of PD-L1 on DC from patients with renal cell cancer, in line with their compromised T-cell induction ability (22). In particular tumor-infiltrating DCs expressing CCR7, indicative of their ability to migrate to TDLN, have been pinpointed as essential for effective PD-1 inhibition (33). Of note, increased PD-1/PD-L1 interactions in TDLN were identified as restraining antitumor T-cell immunity through increased PD-L1 levels on tumor-conditioned DCs (7); PD-L1 blockade resulted in DC-mediated expansion of progenitor-exhausted T cells, which, upon making their way to the tumor, could expand, and differentiate further to mediate antitumor effector functions. These observations provide a compelling argument for combining immune adjuvants, aimed at DC activation and T cell priming, with PD-1 blocking antibodies.

We propose that the lifting of immune suppressive barriers specifically in TDLN may increase the efficacy of ICB through facilitation of the priming and recruitment of new waves of tumor-specific T cells derived from progenitor-exhausted T cells. Indeed, our studies of local intradermal injections in patients with early-stage melanoma, where the primary tumor was removed but TDLN were still accessible to the locally injected immune stimulatory agents, have revealed the singular capacity of TDLN to prime and modulate the systemic antitumor T-cell response (39, 41–43). For CTLA-4 blockade this may entail both increased antitumor effector T-cell activation in the TDLN through CD28-mediated co-stimulation or Treg depletion or inhibition (52–54). In mouse models, we have demonstrated TDLN to also be vital in the efficacy of PD-1 blockade, regardless of local or systemic delivery of therapeutic antibodies (5). Egress of CD8+ effector T cells from TDLN proved vital for subsequent T-cell homing to the tumor and hence for PD-1 blockade efficacy. This finding echoes data we recently obtained from patients with cervical cancer (55): in patients with adenocarcinoma of the cervix there was an apparent accumulation of effector T cells in TDLN, coinciding with decreased frequencies of T cells infiltrating the primary tumor, indicative of faulty egress from the TDLN and homing to the tumor (55). These observations were related to a decreased cDC1-related transcriptional signature in the tumor and an increased Wnt/β-catenin response signature, similar to observations previously reported by Spranger and colleagues in melanoma, showing that β-catenin-mediated restriction in cDC1 recruitment to the tumor stood in the way of effective PD-1 blockade (56). The importance of T-cell trafficking from TDLN to tumor was further underscored by findings from Salmon et al. (8), showing that the absence of cDCs, presenting antigen in TDLN, resulted in a failure of CD8+ T cells to enter the tumor parenchyma after anti-PD-1 treatment, suggesting that increased T-cell infiltration was due to trafficking of T cells previously activated in TDLN. Thus, a picture is emerging of therapeutic PD-1 blockade involving the CD28-mediated expansion of tumor reactive T-cell clones by DCs in TDLN, rather than just the reversal of T-cell exhaustion in the TME. This is consistent with our own observation of superior effects of *in-vitro* PD-1 blockade on HPV16 E6-specific T-cell responses in cervical TDLN over tumors, which was related to the presence of CD8+FoxP3+ T cells with intermediate PD-1 expression levels (30), also previously described by others as prognostically favorable early effectors (18, 57). Such early-effector or progenitor-exhausted T cells can persist for long times in the TDLN in the absence of antigen, are polyfunctional, display a high proliferative capacity and share phenotypic traits with central-memory T cells (58). Upon PD-1 blockade they can efficiently home to the TME and there expand further and differentiate into effector T cells (6, 26). In keeping with this, Chow et al. showed that expression of CXCR3, required for tumor rejection after PD-1 blockade in the MC38 mouse model, was expressed at high levels by progenitor-exhausted or early-effector T cells, whereas it was hardly expressed by terminally exhausted T cells (59).

In conclusion, PD-1 blockade in TDLN can lead to efficient and DC-dependent tumor infiltration by reinvigorated progenitor-exhausted T cells, thus overcoming immune exclusion. In light of these observations, there is a clear rationale for intra- or even peri-tumoral delivery of ICI for optimal access to TDLN (see Figure 1B). Indeed, peritumoral administration ensures optimal access to the tumor's entire and exact catchment area and consequently the most efficient delivery to the greatest number of progenitor-exhausted T cells.

## In Conclusion: The Rise of Local Immunotherapy

Local administration of ICI has been described in pre-clinical models and tested in several types of cancer in clinical trials by us and others, with positive results (42, 59–70). We reported that peritumoral delivery of anti-CTLA-4 in mouse models resulted in an equally efficient antitumor response as observed after systemic administration, without the usually associated inflammatory side effects (65). A recent report from Francis et al. (6) elegantly showed in tumor models that intratumoral administration of CTLA-4 and/or PD-1 blocking antibodies ensured optimal access to TDLN (in contrast to systemic administration) and, moreover, that ipsilateral administration on a site different from the tumor but with lymph drainage to the same lymph node stations afforded equal tumor protection. This is in line with our own observations of the induction of systemic and protective anti-melanoma immunity in early-stage melanoma through local immune modulation of the SLN after surgical removal of the primary tumor, either by CpG-B (44) or by anti-CTLA-4 (71). Of note, systemic treatment with ICI, particularly in early stages of cancer, can result in unacceptably high toxicity. Local administration of lower doses may prove instrumental in limiting this toxicity, while maintaining efficacy by directly targeting the TME and, more importantly, TDLN. Both in breast cancer and melanoma patients it has been well-established that completion lymphadenectomy in case of metastatic involvement of the SLN does not offer any prognostic benefits (72, 73). This notion, together with the recently developed insights that TDLN might be vital for ensuring effective anti-tumor immunity would argue for a neo-adjuvant ICB (or other immunomodulatory) strategy where the lymph nodes in the tumor draining basin are kept in place, possibly even in case of clinically detected lymph node involvement. Our approach of local administration of CpG-B to raise the DC activation state in TDLN and thereby systemic anti-melanoma T-cell immunity, might be used in concert with locally applied ICB to ensure DC-mediated T-cell activation in the TDLN, leading to systemic immunity, allowing new waves of T cells to be recruited to tumors. Indeed, recent reports have shown in patients with advanced melanoma that i.t. administration of CpG (likely ensuring optimal access to TDLN) can lead to increased T-cell infiltration (also of distant non-injected metastases) and even overcome prior resistance to PD-1 blockade (74). Oncolytic virus therapies, such as local treatment with the oncolytic Herpes Simplex virus Talimogene laherperepvec (T-VEC) are, similarly to local injection with TLR agonists, described as belonging to the category of so-called human intratumoral immunotherapy (HIT-IT). T-VEC is an engineered virus, that only replicates in tumor cells and induces secretion of the cytokine GM-CSF from its transgene. Oncolytic viruses can induce local and systemic anti-tumor immune responses through immunogenic cell death induction (75). The local release of GM-CSF results in recruitment and activation of DCs, but likely will also drains to nearby TDLN to activate lymph node resident (LNR)-DCs and promote T cell priming and activation. Moreover, the viral particles contain multiple TLR-ligands, which can directly activate DCs within the TME, but also, when produced and released by dying tumor cells [which in turn will also release damage-associated molecular patterns (DAMPs)], will drain, together with released DAMPs through the lymphatics and activate DCs in TDLN. The OPTiM phase 3 trial, that lead to the approval of T-VEC, compared local T-VEC treatment with systemic GM-CSF treatment and reported improved response rate and also showed signs of enhanced systemic immune responses by tumor regression in non-injected lesions (76). Moreover, enhanced immune cell infiltration upon local T-VEC treatment has been reported in non-injected lesions (77, 78). Combination treatment of local T-VEC with systemic anti-PD-1 therapy was shown to induce high response rates in metastatic melanoma patients (79). Altogether these observations clearly stress the importance of TDLN in immunotherapy efficacy and support the rationale for local delivery of ICI to ensure optimal access to, and modulation of, dysfunctional tumor-specific T cells “lying in wait” in the TDLN, which will subsequently provide systemic tumor control. These considerations have led to a remarkable surge in clinical studies exploring local or i.t. immunotherapy and the publication of a consensus statement on the standardization of terminology and methodologies used in their reporting (80). In time, increased knowledge on the role of TDLN in immunotherapy of cancer will lead to even more rational local therapy strategies in terms of dosing, timing in relation to surgery, and treatment combinations.

## Author Contributions

KP, MF, RV, and TG co-wrote the paper. All authors contributed to the article and approved the submitted version.

## Conflict of Interest

The authors declare that the research was conducted in the absence of any commercial or financial relationships that could be construed as a potential conflict of interest.
